# The effect of mindfulness-based intervention on neurobehavioural functioning and its association with white-matter microstructural changes in preterm young adolescents

**DOI:** 10.1038/s41598-023-29205-8

**Published:** 2023-02-03

**Authors:** Vanessa Siffredi, Maria Chiara Liverani, Dimitri Van De Ville, Lorena G. A. Freitas, Cristina Borradori Tolsa, Petra Susan Hüppi, Russia Ha-Vinh Leuchter

**Affiliations:** 1 Division of Development and Growth, Department of Paediatrics, Gynaecology and Obstetrics, Geneva University Hospitals and University of Geneva, Geneva, Switzerland; 2 Neuro-X Institute, École Polytechnique Fédérale de Lausanne, Geneva, Switzerland; 3 Department of Radiology and Medical Informatics, Faculty of Medicine, University of Geneva, Geneva, Switzerland; 4 SensoriMotor, Affective and Social Development Laboratory, Faculty of Psychology and Educational Sciences, University of Geneva, Geneva, Switzerland; 5 Child Development Lab & Medical Image Processing Lab-Campus Biotech, Chemin Des Mines 9, 1202 Geneva, Switzerland

**Keywords:** Cognitive neuroscience, Human behaviour

## Abstract

Very preterm (VPT) young adolescents are at high risk of executive, behavioural and socio-emotional difficulties. Previous research has shown significant evidence of the benefits of mindfulness-based intervention (MBI) on these abilities. This study aims to assess the association between the effects of MBI on neurobehavioral functioning and changes in white-matter microstructure in VPT young adolescents who completed an 8-week MBI program. Neurobehavioural assessments (i.e., neuropsychological testing, parents- and self-reported questionnaires) and multi-shell diffusion MRI were performed before and after MBI in 32 VPT young adolescents. Combined diffusion tensor imaging (DTI) and neurite orientation dispersion and density imaging (NODDI) measures were extracted on well-defined white matter tracts (TractSeg). A multivariate data-driven approach (partial least squares correlation) was used to explore associations between MBI-related changes on neurobehavioural measures and microstructural changes. The results showed an enhancement of global executive functioning using parent-reported questionnaire after MBI that was associated with a general pattern of increase in fractional anisotropy (FA) and decrease in axonal dispersion (ODI) in white-matter tracts involved in executive processes. Young VPT adolescents with lower gestational age at birth showed the greatest gain in white-matter microstructural changes after MBI.

## Introduction

Children and adolescents born very preterm (VPT; < 32 completed weeks of gestation) are at increased risk for executive, behavioural and socio-emotional impairments that persist into adolescence and adulthood. Executive functioning (EF) is essential for goal-directed and adaptive problem-solving and behaviour. It has been conceptualised in four distinct subdomains: (i) attentional control, (ii) information processing, (iii) cognitive flexibility, and (iv) goal setting^1^. Behavioural and socio-emotional competencies refer to a set of skills related to how individuals identify, express, understand, use and regulate their behaviours as well as their emotions and those of others^2^. Importantly, these competencies are crucial in daily life activities and are closely linked to academic abilities and social behaviours^3–5^. Numerous studies show that white matter alterations are highly common after preterm birth and persist until adolescence and adulthood^6^. Importantly, these white-matter changes have been associated with executive, behavioural and socioemotional deficits in this population^7–9^.

Mindfulness-based intervention (MBI)—commonly defined as the ongoing monitoring of present-moment experience while attending to it in an open and accepting way and without judgment^10^—has been associated with enhanced executive, behavioural and socioemotional functioning in typically developing children and adolescents^11, 12^. In line with behavioural improvements, studies show that MBI may induce structural neuroplastic changes^13^. In this context, diffusion-weighted magnetic resonance imaging (DW-MRI) has been shown to be sensitive to subtle white matter microstructural changes. Fractional anisotropy (FA), derived from the diffusion tensor imaging (DTI) model, has been shown to reflect functionally relevant microstructural properties of white matter, including axonal architecture, extent of myelination and density of axonal fibres comprising axonal bundles^14, 15^. Increased FA measures have been associated with MBI in adult populations in different brain regions and tracts, including frontal regions^16^, callosal regions^17^, anterior cingulate regions^18^, insular^19^, uncinate fasciculus^16^ and superior longitudinal fasciculus^16^. In adolescents, a recent study also showed increased FA in the superior longitudinal fasciculus after a 2-weeks mindfulness video game^20^. To our knowledge, the DTI model is the only methods employed so far to explore potential white-matter microstructural neuroplasticity induced by MBI.

We recently reported a randomised controlled trial showing significant benefits of MBI on executive, behavioural and socio-emotional functioning in VPT young adolescents^21, 22^. The current study aims to assess the association between the benefits of MBI on neurobehavioral functioning and changes in microstructural brain properties. The current study used a pre-post intervention design without a control condition due to limitations of acquiring neuroimaging^22^. Baseline (i.e., pre-MBI) neurobehavioural assessment were conducted in both VPT and full-term young adolescents. The VPT group only completed the MBI. In the VPT group, neurobehavioural measures and questionnaires were collected again post-MBI. VPT young adolescents also completed brain MRI scans at baseline (i.e., pre-MBI) and post-MBI in addition to neurobehavioural measures and questionnaires. To avoid as far as possible test–retest effect in our analyses, we first compared VPT and full-term young adolescents on a range of executive, behavioural and socio-emotional measures. Secondly, we assessed the benefit of an 8-weeks-long MBI on the neurobehavioural measures in which VPT young adolescents showed significant difficulties (i.e., significant reduction in neurobehavioural scores compared to the full-term group). Finally, we explored the association between changes on neurobehavioural measures after MBI and changes on white-matter microstructural measures in the VPT group. As described above, previous findings have revealed changes in quantitative DTI metrics after MBI, specifically an increase in FA. One potential confound of the DTI analyses relates to the inherent non-specificity of the measurement, where observed changes in FA may be due to changes to any combination of axonal dispersion, axonal densities or fibre crossing^23^. In an attempt to provide more specific information about white-matter microstructural changes associated with MBI, a biophysical model of diffusion, NODDI (neurite orientation dispersion and density imaging), was used. The NODDI model allows to distinguish two key variables contributing to changes in FA, including neurite density and fibre orientation dispersion^24^. Combining the high sensitivity of the DTI model with the high specificity of the NODDI model might allow for a better understanding of white-matter microstructural changes associated with changes on neurobehavioural measures after MBI.

## Methods

### Participants

**Figure 1:**
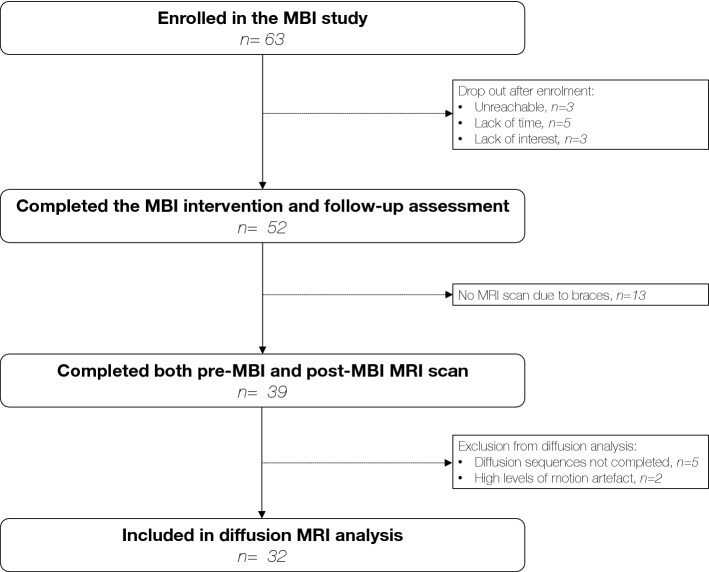
Participant flow chart. This figure shows the number of young adolescents who were enrolled into the MBI study, and of these, the number who had usable scans at both pre- and post-MBI time points and were thus included in the current analysis^21, 22^. MBI, Mindfulness-based intervention; MRI, magnetic resonance imaging.

This study uses data collected as part of the ‘Mindful preterm teens’ study^21, 22^. One hundred and sixty-five VPT young adolescents, i.e., born before 32 gestational weeks, were invited to participate in the study. They were aged 10–14 years born between 01.01.2003 and 31.12.2008 at the Geneva University Hospital, Switzerland, and followed at the Division of Child Development and Growth at the Geneva University Hospital. VPT young adolescents were excluded if they had an intelligence quotient below 70, sensory or physical disabilities (cerebral palsy, blindness, hearing loss), or an insufficient understanding of French. Moreover, some families declined to participate due to lack of time, lack of interest, geographical constraints or unreachability. A total of 63 participants were enrolled in the ‘Mindful preterm teens’ study and 52 of them completed the MBI and neurobehavioural assessment before and after MBI. Of the 52 young adolescents, 39 completed MRI scans before and after intervention, and 32 were included in the current diffusion MRI analyses (diffusion sequences not completed, n = 5; high level of motion artefacts, n = 2), Fig. 1.

Moreover, 22 term-born young adolescents aged between 10 and 14 years old were recruited through the community and completed a neurobehavioural assessment similar to the VPT group prior to the MBI. The term-born group did not complete the MBI.

All experimental protocols were approved by the Swiss Ethics Committees on research involving humans, ID: 2015-00175. All methods were carried out in accordance with relevant guidelines and regulations. Written informed consent was obtained from primary caregivers and participants.

### Procedure

**Figure 2:**
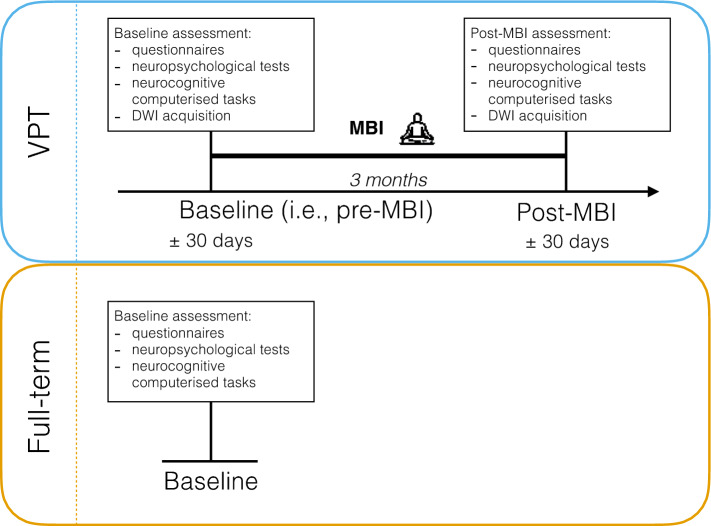
Illustration of the pre-post intervention design of the current study^22^. The VPT group is represented in blue and the full-term control group is represented in orange.

The current study is part of a larger randomised controlled trial conducted in VPT young adolescents to evaluate the effect of an MBI on executive, behavioural and socio-emotional functioning^21, 22^. The current study used a pre-post intervention design without a control condition due to limitations of acquiring neuroimaging in VPT young adolescents^22^; and baseline neurobehavioural assessment was conducted in full-term young adolescents, see Fig. 2 for an illustration.

### Mindfulness-based intervention

The proposed MBI was designed by the authors and detailed in a previous study^22^, adapting well-known MBI programs, including Mindfulness-Based Stress Reduction (MBSR^10^) and Mindfulness-Based Cognitive Therapy (MBCT^25^), to adolescents’ needs. Adaptation to youth, besides language and instructors' more active attitude, included shorter sessions, more guided (with less silence) and shorter mediation practices (between 2 and 10 min). The program consisted of 8 weekly sessions in groups of up to 8 participants, lasting 1h30. Two instructors were present for each group throughout the intervention. The instructors were certified MBI instructors with a long-term training in mindfulness (RHVL & AM: MBCT certification; FSG: MBSR certification; MMS: ongoing MBSR certification^22^), with at least one instructor per session being either a paediatrician or a child psychologist. For each session one theme was addressed, such as attention and the stabilisation of the focus of attention, bodily sensations, breath, emotions, thoughts, compassion, stress, stress reactivity and coping strategies. Different formal meditation practices were introduced, such as sitting mediation with different objects of attention, body scan, walking meditation and mindful movement. Participants had the opportunity to share experiences with their peers and the instructors during each session, allowing the recognition of individual patterns of behaviour, and at the same time, the appreciation of the shared experiences as a community. Compared to the MBSR and MBCT programs, all formal practices where shorter. We also added playful practices—such as a card game needing very quick responses to win the game-with the objective of helping young adolescents to become aware of their bodily reactions to emotions. They were also invited to practice between 5 to 20 min per day at home, using guided mediation recorded by the instructors. The details about each session can be visualised in Supplementary Table S2 (see Ref.^22^ for further details). The same MBI was already used with success at the Geneva University Hospital in the context of another study^26^.

Regarding adherence and compliance with the MBI in the context of the randomised controlled trial^22^ of the young adolescents who were initially screened (n = 165), the final participation rate in the randomised controlled trial was 38.2%, with 31.5% of participants who completed MBI and follow up assessments. Once the participants started the MBI program, there was no drop-out; showing that participants largely accepted the MBI program (see Siffredi et al. for further details on the MBI^22^)”.

### General intelligence measure

The Wechsler Intelligence Scale for Children—4th Edition (WISC-IV^27^; was used to evaluate the General ability index (GAI) as a measure of general intellectual functioning. The GAI is derived from the core verbal comprehension and perceptual reasoning subtests (mean = 100, SD = 15).

### Neurobehavioral outcome measures

Participants’ executive, behavioural and socio-emotional functioning were assessed using parent-report and self-report questionnaires, neuropsychological testing and computerised neurocognitive tasks, see Supplementary Table S1.

(i) Executive function measures

Behaviours related to executive function of young adolescents were assessed using the Behaviour Rating Inventory of Executive Function-parent version (BRIEF)^28^. The BRIEF gives two standardised subscales, the Behavioural Regulation Index (BRI) and the Metacognition Index (MI) as well as a global score called the Global Executive Composite (GEC). Neurocognitive computerised tasks comprised: (i) the Flanker Visual Filtering Task, in which reaction time of the congruent condition was used to assess speed of processing (which belongs to the information processing subdomain), and the inhibition score (reaction time in incongruent conditions—reaction time in congruent conditions) was used as a measure of the subdomain of attentional control^1^; (ii) the child-adapted version of the Reality Filtering task, in which the temporal context confusion index (TCC) was used as a reality filtering measure, which involves integration of different executive processes^29, 30^. Neuropsychological testing included the Letter-Number Sequencing subtest from the Wechsler Intelligence Scale for Children, 4th Edition (WISC-IV) assessing working memory, which belongs to the cognitive flexibility subdomain^1^. Given the strong association between executive functions and mathematical abilities in children and adolescents^31^, we also used the total score of the Tempo Test Rekenen to assess timed mathematical achievement^32^.

(ii) Behavioural and socio-emotional competencies measures

The total score of the Strength and Difficulties Questionnaire—parent version (SDQ) was used to assess behaviour in daily life^33^. Participants completed three self-reported questionnaires: the KIDSCREEN-27 items questionnaire was used to assess the quality of life of the participants^34^; the total score of the Social Goal Scale was used to assess social responsiveness and social relationships^35^; and the total score of the Self-Compassion Scale—Short form was used to assess the main components of self-compassion^36^. Neuropsychological testing included the Affect Recognition subtest (A Developmental Neuropsychological Assessment, 2nd Edition—NEPSY-II), giving a total score assessing facial emotional recognition and the Theory of Mind subtest (NEPSY-II), giving a total score measuring the ability to understand mental functions, such as belief, intention or deception^37^.

### Magnetic resonance imaging

MRI data were acquired at Campus Biotech in Geneva, Switzerland, using a Siemens 3 T Magnetom Prisma scanner. All participants completed a simulated “mock” MRI session prior to their first MRI scan. This preparation process was conducted by trained research staff and allowed participants to familiarise themselves with the scanner and the scanning process, eventually raising any concerns they might have had prior to the MRI scan. Furthermore, this process is known to facilitate the acquisition of good quality MRI images in children and adolescents^38^.

A multi-shell diffusion‐weighted (DW) echo planar imaging (EPI) protocol was used and included four shells. The first sequence, referred to as ‘*b200*’, included 10 gradient directions with b‐values of 200 s/mm^2^; the second one referred to as ‘*b1700*’, included 30 gradient directions with b‐values of 1700 s/mm^2^; the third one referred to as ‘*b4200a*’, included 26 gradient directions with b‐values of 4200 s/mm^2^; and the fourth one referred to as ‘*b4200b*’, included 24 gradient directions with b‐values of 4200 s/mm^2^. Each of four sequences included the acquisition of 4 images with b‐value = 0 s/mm^2^ images, and the parameter set for all sequences were: TR = 7000 ms, TE = 87 ms, FOV = 234 × 243 mm, slice thickness = 1.3 mm, voxel size = 1.3 × 1.3 × 1.3 mm.

### Diffusion image preprocessing

Visual inspection of raw data for brain coverage, spike artefacts, severe head motion, and other severe image artefacts was completed and participants were excluded if necessary. The four diffusion shells (*b200, b1700, b4200a, b4200b*) were preprocessed independently using MRtrix3^39^ and using the following pipeline: (a) denoising, (b) Gibbs ringing removal, (c) correction for movement and eddy current‐induced geometric distortions using the eddy tool implemented in FSL^40^. The first b = 0 s/mm^2^ images of the *b1700, b4200a, b4200b* sequences were linearly registered to the first b = 0 s/mm^2^ image of the *b200* sequence using FreeSurfer to bring them into *b200* space before merging them together. The brain extraction tool (BET) from FSL was then applied to the combined *b200, b1700, b4200a, b4200b* image to remove non-brain tissue and subsequently intensity normalisation was applied. Following Pines et al. recommendations^41^, the resulting multi-shell diffusion weighted image was then used for both DTI and NODDI models fitting and tractography.

### Diffusion models fitting

The DTI model was applied to the resulting multi-shell diffusion weighted image and whole-brain maps of FA was calculated for each participant. FA (between 0 and 1) is a measure of the directionality of diffusion that characterise the variance of the three eigenvalues pairs that represent the direction and magnitude of diffusivity along the three orthogonal axes (ν1, λ1; ν2, λ2; ν3, λ3)^42^. In addition, intra-cellular volume fraction and orientation dispersion indices were estimated from the resulting multi-shell diffusion weighted image using the NODDI model^24^. The NODDI Matlab Toolbox http://www.nitrc.org/projects/noddi_toolbox was used to extract maps of neurite density index (NDI) and fibre orientation dispersion (ODI) across the brain for each participant.

### Tractography and tractometry measures

**Figure 3:**
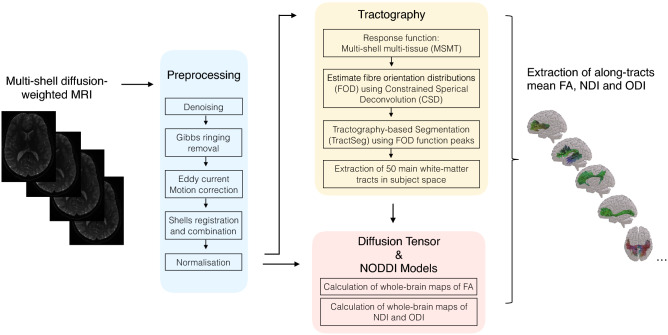
Flowchart summarizing the computation and analysis of microstructural properties of white-matter tracts. Briefly, diffusion-weighted images were preprocessed. Whole-brain multi-shell multi-tissue constrained spherical deconvolution (MSMT-CSD) was extracted and fibre orientation distribution (FOD) function peaks map were input into Tractography-based Segmentation (TractSeg) to extract the main white-matter tracts in subject space. Mean fractional anisotropy (FA), neurite density index (NDI) and orientation dispersion index (ODI) were then extracted along each track by applying the diffusion tensor and NODDI models.

Whole-brain fibre orientation distributions (FOD) were estimated using the multi-shell multi-tissue constrained spherical deconvolution (MSMT-CSD) method^43^, resulting in a condensed representation of diffusion along three principal fibre directions per voxel according to tissue type (grey, white, cortico-spinal fluid). Tractography-based Segmentation (TractSeg) use a supervised-learning approach with a aconvolutional neural network-based that directly segments tracts in the field of fibre orientation distribution function (fODF) peaks without using parcellation^44^. TractSeg has achieved state-of-the-art performance and allows for an accurate reconstruction of fibre tracts in subject space, thus avoiding the problem of inaccurate coregistration of tracts with varying size and shape. Whole-brain fibre orientation distribution function (fODF) peaks map were input into a two stage fully convolutional neural network trained using segmented priors of 72 anatomically well-defined white matter tracts from the Human Connectome Project. Using the tractometry function, along-tract mean FA, NDI and ODI were calculated for the 50 most consistent white-matter tracts in each subject’s native space, see Fig. 3.

### Statistical analyses

Statistical analyses were performed using R version 4.0.3^45^ and RStudio version 1.3.1093^46^.

In the context of this pre-post intervention design (i.e., both the VPT and full-term young adolescents completed baseline neurobehavioural assessment; only the VPT group completed the MBI with baseline (i.e., pre-MBI) and post-MBI neurobehavioural assessment and DWI acquisition), we employing the following methods:

- Neurobehavioural functioning: comparison of the VPT and full-term groups

Group comparisons between the preterm and the full-term groups were conducted for all neurobehavioural measures using independent-sample t-test, given that assumptions for parametric testing were violated. P values were corrected for multiple comparisons with the false discovery rate (FDR, q values < 0.05^47^). Effect size were assessed using Wilcoxon effect size (r).

- Impact of MBI on neurobehavioral functioning affected by preterm-birth

Paired-sample t-test were used to evaluate the impact of MBI on the neurobehavioural measures showing a significant deficit in the VPT group as compared to the full-term group. P values were corrected for multiple comparisons with the false discovery rate (FDR, q values < 0.05^47^). Effect size were assessed using Wilcoxon effect size (r).

- Association between neurobehavioural changes and microstructural changes after MBI

For each neurobehavioural and mean microstructural measures (i.e., FA, NDI and ODI), score differences between assessment at pre-MBI and assessment at post-MBI were calculated for each participant. Score differences for neurobehavioural measures will be referred to as Δ neurobehavioural measures (neurobehavioural score post-MBI–neurobehavioural score pre-MBI). Score differences for mean FA, NDI and ODI measures will be referred to as Δ mean FA, NDI and ODI measures (i.e., mean FA post-MBI–mean FA pre-MBI; mean NDI post-MBI–mean NDI pre-MBI; mean ODI post-MBI–mean ODI pre-MBI). Negative Δ indicates a reduction of the scores between two time points, whereas positive Δ indicates an increase in scores between two time points.

Partial least square correlation analyses (PLSC) were performed to evaluate associations between changes in neurobehavioural measures and changes in mean microstructural measures after MBI. PLSC is a data-driven multivariate technique that maximizes the covariance between two matrices by identifying latent components (LCs) which are linear combinations of the two matrices, i.e., Δ neurobehavioural functioning measures and Δ mean microstructural measures^48^. A publicly available Matlab PLSC implementation was used: https://github.com/danizoeller/myPLS^49, 50^.

In a first PLSC, associations between changes in neurobehavioural measures and changes in mean FA measures after MBI were investigated to capture any changes related to white-matter microstructural properties measured by the classical tensor model.

The neurobehavioural functioning data refers to the three Δ neurobehavioural measures showing significant difference between the full-term and VPT group (i.e., BRIEF MI, BRIEF GEC, SDQ total), as well as age at assessment and gestational age at birth. The neurobehavioural functioning data were stored in a 32 × 5 matrix denoted X. Each row of X represents one subject and the matrix's 5 columns are made up of the three Δ neurobehavioural measures showing significant difference between the full-term and VPT group, as well as age at assessment and gestational age at birth. The Δ mean FA data were gathered in a 32 × 50 matrix denoted Y, with each row matching one subject and each column one Δ mean FA measure for each of the 50 tracts extracted using the TractSeg framework. A cross-covariance matrix (which is effectively a correlation matrix, since the data are z-scores) was then computed between X and Y. Singular value decomposition was then applied to this cross-covariance matrix, resulting in latent components. Each latent component is composed of neurobehavioural functioning saliences and Δ mean FA saliences, and saliences indicate how strongly each neurobehavioural functioning measures and Δ mean FA measures contribute to the multivariate association of neurobehavioral functioning and Δ mean FA. The significance of latent components was determined by permutation testing (1000 permutations). Stability of neurobehavioural functioning saliences and Δ mean FA saliences were estimated using bootstrapping (500 bootstrap samples with replacement). Bootstrap ratio z-scores for each neurobehavioural functioning and Δ mean FA measures were obtained by dividing each neurobehavioural functioning and Δ mean FA salience by its bootstrap-estimated standard deviation, and a p-value was obtained for each bootstrap ratio z-score. Following the PLSC interpretation^51^, the contribution of neurobehavioural functioning and Δ mean FA saliences for a given latent component was considered robust at p < 0.01 (i.e., absolute bootstrap ratio z-scores above 2.3 or below − 2.3).

In a second PLSC, we investigated further associations between changes in neurobehavioural measures and changes in microstructural measures using the NODDI model, including mean NDI and ODI measures after MBI. A procedure similar to the first PLSC was employed using a 32 × 5 matrix denoted X containing the neurobehavioural functioning data and a 32 × 100 matrix denoted Y containing the Δ mean NDI and Δ mean ODI.

## Results

### Participant characteristics

The final sample included 32 VPT and 22 full-term young adolescents between 10 and 14 years of age. Baseline characteristics were similar between VPT and full-term participants for sex, age at the assessment and socio-economic status. The VPT group had significantly lower general ability index compared to the full-term group, see Table 1.

**Table 1:** Neonatal and demographic characteristics of the VPT and full-term participants.

	VPT (n = 32)	Full-term (n = 22)	Group comparison
Birth weight Mean (SD) in grams	1240 (416.2)	3403 (451.1)	t(42.832) = − 17.864, p < 0.001
Gestational age Mean (SD) in weeks	29.2 (1.9)	39.8 (1.5)	t(50.56) = − 22.481, p < 0.001
Age at assessment Mean (SD) in years	12.2 (1.2)	11.9 (1.1)	t(48.75) = 0.844, p = 0.403
Sex, n	15 females 17 males	9 females 13 males	*X*^2^ (1, *N* = 55) = 0.188, *p* = 0.665
Socio-economic status Mean (SD)	4 (2.7)	3.1 (1.4)	t(49.34) = 1.685, p = 0.098
General ability index (GAI) mean (SD)	109.3 (10.6)	116.4 (11.4)	t(43.25) = − 2.288, p = 0.027

For VPT young adolescent, age correspond to the age at pre-MBI assessment. General ability index (GAI) was evaluated using the Wechsler Intelligence Scale for Children—4th Edition, WISC-IV^27^ as a measure of general intellectual functioning (mean for the general population = 100, standard-deviation = 15). Socioeconomic status of the parents was estimated using the Largo scale, a validated 12-point score based on maternal education and paternal occupation^70^. Higher socio-economic status scores reflect lower socio-economic level. Independent-sample t-test or Chi-square as appropriate were used to compare the preterm and full-term participants.

Detailed neonatal characteristics of the VPT group are provided in Table 2. None of the VPT young adolescents have a history of brain injury. There was a significant negative association between both birth weight (r = − 0.88, p < 0.001) and gestational age (r = − 0.89, p < 0.001) with the length of hospitalisation at birth. Similarly, there was a significant negative association between both birth weight (r = − 0.41, p = 0.021) and gestational age (r = − 0.52, p = 0.002) with bronchopulmonary dysplasia (BPD) at birth. This indicated that higher level of prematurity in VPT young adolescents were associated with longer length of hospitalisation at birth and increased risk of BPD.

**Table 2:** Detailed neonatal characteristics of the VPT group.

	VPT (n = 32)
Birth weight, mean (SD) in grams	1240 (416.2)
Gestational age, mean (SD)in weeks	29.2 (1.9)
Head circumference, mean (SD) in cm	26.3 (3.1)
Length of hospitalisation, mean (SD) in days	57.9 (29.8)
Multiple births, n (%)	10 (31.3%)
cPVL, n (%)	0 (0%)
IVH—Grades III and IV, n (%)	0 (0%)
BPD, n (%)	10 (31.3%)

*cPVL* Cystic periventricular leukomalacia, *IVH* intraventricular haemorrhage, *BPD* bronchopulmonary dysplasia.

### Neurobehavioural outcomes: comparison of the VPT and full-term groups

**Figure 4:**
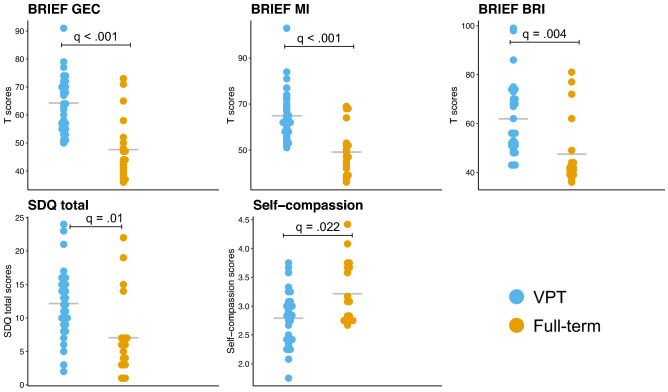
Neurobehavioural scores showing significant group differences between the VPT (prior to the MBI) and the full-term groups using independent sample t-test (FDR, q values < 0.05). The mean for each measure and each group is represented by a grey line.

Neurobehavioural measures showing a significant difference and surviving FDR correction between the VPT and the full-term groups are presented in Fig. 3. The VPT group showed higher BRIEF GEC (q < 0.001), BRIEF MI (q < 0.001), BRIEF BRI (q < 0.001) and SDQ total scores (q < 0.001) reflecting more executive function and behavioural difficulties in daily life compared to the full-term group. Moreover, the self-compassion score was reduced in the VPT group compared to the full-term group (q < 0.011), see Fig. 4 and Supplementary Table S3. The other neurobehavioural scores were comparable between the VPT and full-term groups.

### Impact of MBI on neurobehavioral measures affected by preterm-birth

**Figure 5:**
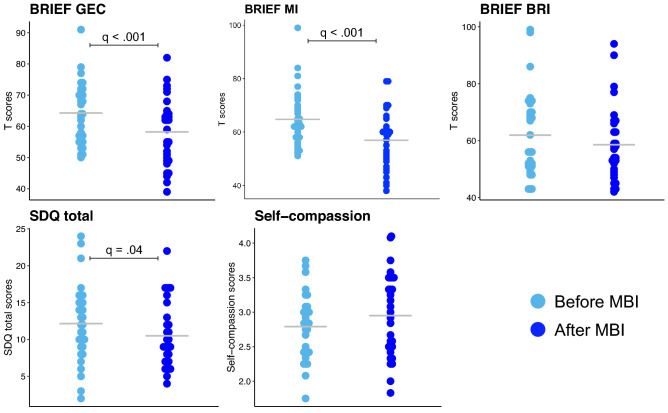
Neurobehavioural scores before and after MBI. Paired-sample t-tests was used to compared scores before and after MBI (FDR, q values < 0.05). The mean for each measure and each group is represented by a grey line.

A significant decrease after MBI was observed for the following measures: BRIEF GEC (q < 0.00), BRIEF MI (q < 0.001) and SDQ total scores (q < 0.04), reflecting significantly less executive and behavioural difficulties in daily life. There was no significant difference before and after MBI for the BRIEF BRI and self-compassion scores, see Fig. 5 and Supplementary Table S4.

### Association between neurobehavioural changes and microstructural changes after MBI

**Figure 6:**
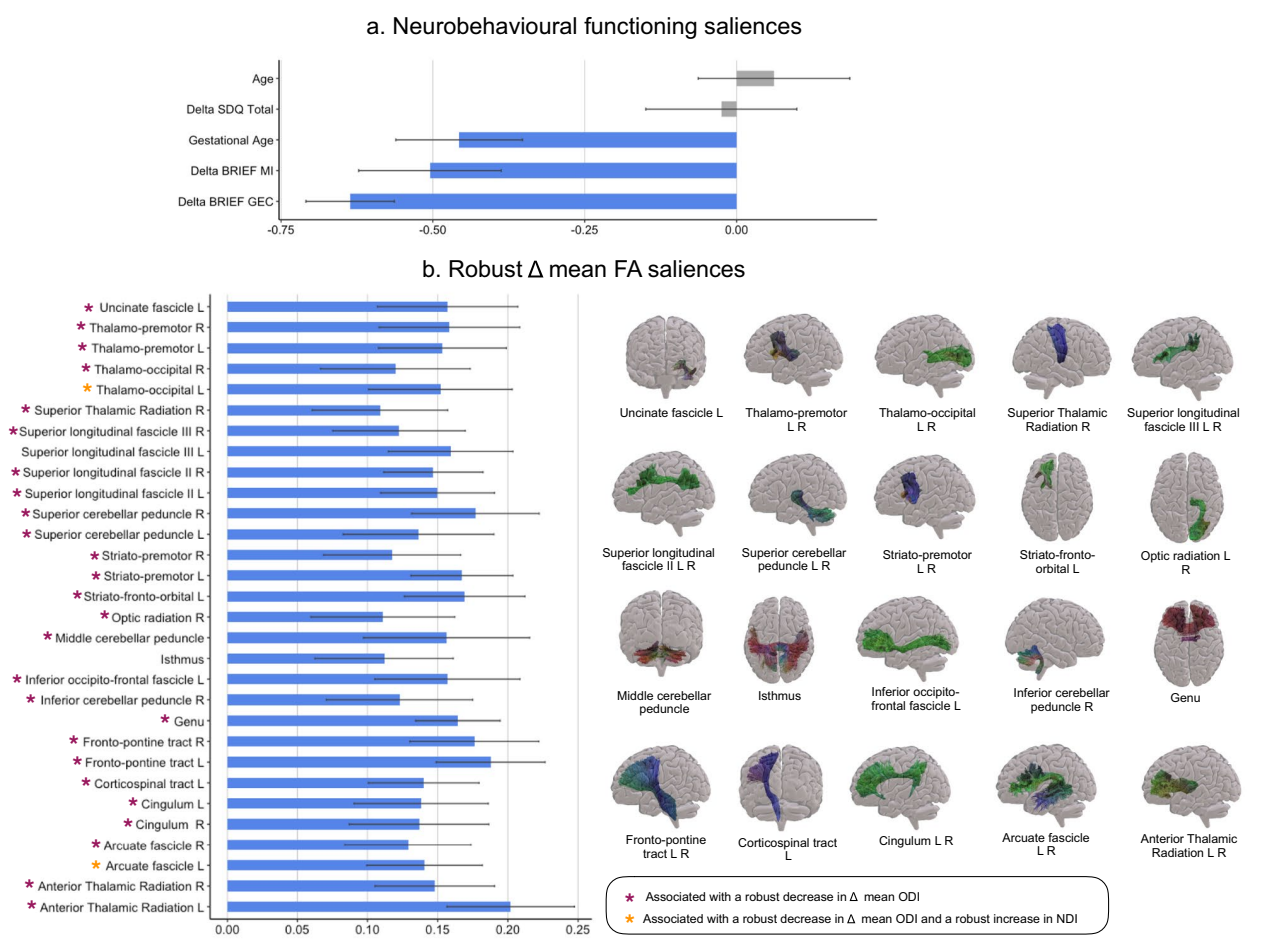
Associations between neurobehavioural functioning and Δ mean FA and Δ mean ODI based on the PLSC analysis. (**A**) Neurobehavioural functioning saliences: the diverging graph show mean saliences averaged across bootstrap samples and their bootstrap-estimated standard deviations (x-axis) for each neurobehavioural functioning measure (y-axis); robust saliences are represented in blue. Of note, saliences bellow 0 indicate a decreased in scores after MBI and saliences above 0 indicate an increased in scores after MBI. (**B**) Robust Δ mean FA saliences: the diverging graph show robust mean saliences averaged across bootstrap samples and their bootstrap-estimated standard deviations (x-axis) for each Δ mean FA along a given tract (y-axis). The tracts extracted from TractSeg and showing robust Δ mean FA saliences are shown on the right side and the colour code for principal direction of diffusion is used: red (left–right), green (anterior–posterior), blue (inferior–superior). Based on the results from the second PLSC analyses: pink star indicates a robust negative contribution of Δ mean ODI along the given tract associated with a similar pattern of neurobehavioural functioning saliences; and orange star indicates both a robust negative contribution of Δ mean ODI and a robust positive contribution of Δ mean NDI along the given tract associated with a similar pattern of neurobehavioural functioning saliences. Of note, saliences below 0 indicate a decreased in scores after MBI and saliences above 0 indicate an increased in scores after MBI; L left, R right.

The first PLSC analysis applied on neurobehavioural functioning (i.e., Δ neurobehavioural measures, age at the assessment and gestational age) and Δ mean FA identified one statistically significant latent component: latent component 1 (p = 0.005). Mean saliences as well as their bootstrap-estimated standard deviations for neurobehavioural functioning and Δ mean FA are reported in Supplementary Table S5. Latent component 1 revealed a general pattern of association between neurobehavioural functioning and Δ mean FA, see Fig. 6. A decrease in Δ scores of the BRIEF GEC and BRIEF MI were associated with an increase in Δ mean FA values for a range of tracts, including the arcuate fascicle left/right, the anterior thalamic radiation left/right, the superior thalamic radiation right, the genu and isthmus of the corpus callosum, the cingulum left/right, the corticospinal tract left, the fronto-pontine tract left/right, the inferior cerebellar peduncle right, the inferior occipito-frontal fascicle left, the middle cerebellar peduncle, the optic radiation right, the superior cerebellar peduncle left/right, the superior longitudinal fascicle II and III left/right, the uncinate fascicle left, the thalamo-premotor and thalamo-occipital tract left/right, the striato-fronto-orbital left as well as the striato-premotor tract left/right. Moreover, lower gestational age was associated with a robust increase in Δ mean FA values in these same tracts, see Fig. 6a. Importantly these microstructural changes were not associated with the age at the assessment of the participants.

The second PLSC analysis applied on neurobehavioural functioning (i.e., Δ neurobehavioural measures, age at the assessment and gestational age) and Δ mean NDI and ODI identified one statistically significant latent component: latent component 1 (p = 0.02). Mean saliences as well as their bootstrap-estimated standard deviations for neurobehavioural functioning and Δ mean NDI and ODI are reported in Supplementary Table S6 and Supplementary Fig. S1. Latent component 1 revealed an association between decrease in Δ scores of the BRIEF GEC and BRIEF MI with a general decrease in Δ mean ODI for all tracts showing a robust increase in Δ mean FA, including: arcuate fascicle left/right, the anterior thalamic radiation left/right, the superior thalamic radiation right, the genu of the corpus callosum, the cingulum left/right, the corticospinal tract left, the fronto-pontine tract left/right, the inferior cerebellar peduncle right, the inferior occipito-frontal fascicle left, the middle cerebellar peduncle, the optic radiation right, the superior cerebellar peduncle left/right, the superior longitudinal fascicle II left/right and III right, the uncinate fascicle left, the thalamo-premotor and thalamo-occipital tract left/right, the striato-fronto-orbital left as well as the striato-premotor tract left/right; with the exception of the isthmus of the corpus callosum and the superior longitudinal fascicle III left, see Fig. 6. This decreased in Δ scores of the BRIEF GEC and BRIEF MI were also associated with a combined increased in Δ mean NDI and decrease in Δ mean ODI for left-lateralised tracts including: the arcuate fascicle, inferior cerebellar peduncle, thalamo-parietal and thalamo-occipital. Moreover, the decreased in Δ scores of the BRIEF GEC and BRIEF MI were associated with a robust reduction of ODI only on the rostrum of the corpus callosum, on the superior longitudinal fascicle I left and on the striato-fronto-occipital right. Finally, the general increase in Δ NDI and decrease in ODI measures were also associated with higher gestational age in VPT young adolescents. Importantly these microstructural changes were not associated with the age of the participants.

## Discussion

In this study, we investigated the benefits of an 8-weeks-long MBI on neurobehavioral functioning and its association with white-matter microstructural changes in VPT young adolescents using both DTI and NODDI parameters. Our finding showed an enhancement of global executive functioning in daily life after MBI that was associated with a general pattern of significant increase in FA along with a decrease in ODI in a range of white-matter tracts involved in executive processes. This general pattern of increase FA and decrease ODI values was also negatively associated with gestational age at birth, meaning that the microstructural changes in FA and ODI after MBI was particularly marked in young adolescents with lower gestational age. Despite promising findings, it is worth mentioning that the current study did not include a control or placebo intervention. Future studies would be needed to address this point.

In the present study, VPT young adolescents showed significant difficulties in executive and behavioural functioning in daily life as measured by the BRIEF and SDQ parent questionnaires, as well as significantly lower self-compassion using a self-reported questionnaire compared to full-term controls. These weaknesses have been previously described in several cohorts of children, adolescents and adults born prematurely^52^. After an 8-weeks MBI, an enhancement of executive and behavioural functioning in daily life of VPT young adolescents was observed. These behavioural results corroborate the results from our previous study using a gold standard randomised controlled trial design^21^, which indicates that the gain observed after MBI on these executive and behavioural measures is independent of test–retest effect. The benefits of MBI observed on daily executive and behavioural functioning are also consistent with previous studies completed in other populations^11^.

Combining both the classical DTI model and the NODDI model (i.e., a multi-component model that captures neurite morphology), microstructural changes associated with the beneficial effect of MBI on executive and behavioural functioning were explored. Our finding show that the gain observed in executive functioning after an 8-week MBI was associated with a general pattern of increase in FA along with a decrease in ODI. While an increase in FA values reflect changes in the diffusion of water molecules along a given tract, the decrease in axonal dispersion indicate more specifically an enhancement in the coherence of the spatial organisation of the axons^53^. This pattern of higher FA coupled with decrease ODI in a specific white-matter tract is commonly interpreted as an indication of increased connectivity^54^. Importantly, this pattern of microstructural changes associated with improved executive functioning after MBI was observed in white-matter tracts known to be involved in different executive processes, i.e., attentional control, inhibition, processing speed, updating, fluency and self-regulation^55–57^ or in white-matter tracts implicated in sensorimotor circuits that support executive functioning^58^, including the anterior and superior thalamic radiation, the genu of the corpus callosum, the cingulum, the fronto-pontine tract, the inferior occipito-frontal fascicle, the superior longitudinal fascicle II and III, the arcuate fascicle, the thalamo-premotor and thalamo-occipital tract, the striato-fronto-orbital as well as the striato-premotor tract. Moreover, the uncinate fascicle, known to be involved in emotional-regulation processes, also showed this pattern of increase FA and reduced ODI associated with improvement in executive functioning after MBI^59, 60^. These results corroborate previous research studies showing that the practice of MBI or MBI-related approaches such as meditation are associated with increases in FA in these tracts or related-regions of interest^16–18, 20, 54, 61, 62^. Moreover, there was an association between changes in executive functioning after MBI with changes in cerebellar mean FA including, the inferior cerebellar peduncle, the middle cerebellar peduncle and the superior cerebellar peduncles. Again, these FA changes were more specifically coupled with a decrease in neurite orientation dispersion. Cerebellar regions have been implicated in executive functions and proposed to play a role of “control of behaviour” by linking movement to thought^63, 64^. This is also in line with previous results showing changes in grey matter density of cerebellar regions after an 8-week Mindfulness program^65^. Compared to previous findings, the use of the NODDI model in the current study allows to characterise more precisely this increase in FA explained by a concurrent decrease in neurite orientation dispersion.

Altogether, the enhancement in executive functioning was not only associated with FA changes in white-matter tracts involved in executive functioning per se; but it was also associated with FA changes in white-matter tracts implicated in bottom-up and sensorimotor interaction on which executive functions rely. Importantly, the NODDI model allowed to characterise more precisely this general increase in FA with concurrent decrease in neurite orientation dispersion. As opposed to executive functioning, the changes observed in socio-emotional and behavioural functioning after MBI, as measured by the SDQ total score, were not significantly associated with changes in mean FA.

Additionally, the increase in FA and reduction in ODI values following MBI of these tracts was associated with lower gestational age at birth. In other words, the microstructural gain observed on the tracts mentioned above was bigger in the most preterm young adolescents. It is likely that MBI is most beneficial for the most vulnerable young adolescents as they have more opportunities for further enhancements. This is consistent with the results of our randomised controlled trial showing individual variability in the response to MBI with a greater benefit on overall executive skills in VPT young adolescents born with higher risk (i.e., smaller gestational age and lower weight at birth), in comparison with VPT young adolescents born with lower risk^21^. This pattern of increased benefit of MBI in the most vulnerable individuals has already been observed in children with weaker executive abilities^66^.

Finally, the reduction in executive difficulties observed after an 8-week MBI was also associated with an increase in NDI in white-matter tracts implicated in executive functioning, including the arcuate fasciculus as well as thalamic and cerebellar tracts. This pattern reflecting an increase in packing density of axons in white matter in a few tracts was also associated with reduced gestational age at birth. These results go in line with the suggested increased benefit of MBI in the most vulnerable individuals and in individuals with weaker executive abilities^21, 66^.

Our findings should also be considered in light of the limitations of the current study. Firstly, despite being part of a larger randomised controlled trial^21, 22^, this study uses a pre-post intervention design in the VPT group who completed the MBI, including pre-post MBI neurobehavioural assessments and DWI acquisitions. Therefore, the absence of an active control condition or a placebo condition is an important limitation to this study. Comparing MBI to an engaging active control condition would be necessary to provide reliable evaluation of the impact of an MBI in young VPT adolescents on behavioural and related changes in white-matter microstructural properties. Secondly, mindfulness practice at home was not recorded in our study. We were therefore unable to have quantitative data on actual home practice, an issue faced by many interventional studies. For future research, it might be beneficial to collect precise quantitative data on their practice of mindfulness at home. For example, a phone application in which participants can access guided meditations and that is able to register data on individual usage might be a more reliable tool in young adolescents. Thirdly, we observed executive, behavioural and socio-emotional difficulties in VPT young adolescents compared to full-term controls measured via self- and parent-reported questionnaires only. It is possible that neurobehavioural tests used in this study were not able to capture everyday life difficulties experienced by these young adolescents. This point is closely related to the concept of ecological validity of the neurobehavioural tests used^67^. Similarly, the beneficial effect of MBI on executive and socio-emotional competencies were observed through parent-questionnaires. These changes observed via parents-reported questionnaires might be questionable as parents were not blind to the intervention. This point might be methodologically improved via the inclusion of an active control condition or placebo condition, as done in previous studies^68, 69^. Nevertheless, the association found with micro-structural changes on specific tracks suggest that the benefit of MBI observed via parent questionnaire are well-grounded.

## Conclusions

This study provides evidence that the enhancement in executive functioning after an MBI in VPT young adolescents is associated with white-matter microstructural changes in tracts involved in executive processes as well as in tracts involved in sensorimotor circuit supporting and interacting with executive processes. Our study also suggests that the most vulnerable VPT young adolescents, i.e., the ones with lowest gestational age at birth, show the biggest gain in terms of white-matter microstructural changes. Finally, MBI appears to be a promising tool for enhancing executive functioning and white-matter brain plasticity in a vulnerable population such as VPT young adolescents.

## Supplementary Information

Supplementary Information.

## Data Availability

Deidentified individual participant data (including data dictionaries) will be made available, in addition to study protocols, the statistical analysis plan, and the informed consent form. The data will be made available upon publication to researchers who provide a methodologically sound proposal for use in achieving the goals of the approved proposal. Proposals should be submitted to Russia.HaVinhLeuchter@unige.ch.
