# Supplementary material for: The effect of mindfulness-based intervention on neurobehavioural functioning and its association with white-matter microstructural changes in preterm young adolescents
Source: Sci Rep. 2023 Feb 3;13:2010. doi: 10.1038/s41598-023-29205-8 (PMC9898533; doi:10.1038/s41598-023-29205-8)
Supplement: Supplementary file 1 — Supplementary Information. [file 41598_2023_29205_MOESM1_ESM.pdf]

Supplementary Materials

Supplementary Tables

Supplementary Table S1. Details of the neurobehavioural outcome measures and scores

Domains	Modalities	Measures	Description	Scores
**Executive competences**				
	Parent questionnaire			
		Behaviour Rating Inventory of Executive Function, parent version (BRIEF, Gioia, et al. [1])		
			The BRIEF parent questionnaire provides an index of attention, hyperactivity and impulsivity in everyday life. The BRIEF comprises 86 items over two standardised subscales: (i) Behavioural Regulation Index (BRI) comprising 3 subscores including, Inhibit, Shift, Emotional Control; (ii) Metacognition index (MI) comprising 5 subscores including, Initiate, Working Memory, Plan/Organise, Organisation of Materials, Monitor; as well as a global score called the Global Executive Composite (GEC). Higher scores reflect increased difficulties in executive functioning (M=50; SD=10).	BRIEF GEC<br>BRIEF BRI<br>BRIEF MI
	Neuropsychological tests			
		Letter-Number Sequencing (WISC-IV, Wechsler [2])		
			The letter-number sequencing is a working memory task. Sequences of number and letters are read to the participant, and he/she is then asked to re-sequence the numbers in numerical order from lowest to highest and then to sequence the letters in alphabetical order. Standardised scores were used (M=10; SD=3). Higher standardised scores reflect higher working memory skills.	Letter-number sequencing
		Tempo Test Rekenen [3]		
			The Tempo Test Rekenen is an arithmetic test consisting of 200 arithmetic number fact problems presented in five rows (one row with addition, one row with subtraction, one row with division, one row with multiplication, and one mixed problem row). Within each row, the problems increase in difficulty. Participant are asked to solve as many items as possible within 1 min per row. The total raw score was age-adjusted for each participant using the procedure described in the main statistical analyses section. Higher total scores reflect higher arithmetic skills.	Tempo test
	Neurocognitive computerised tasks			
		Flanker Visual Filtering Task [4]		
			The Flanker Visual Filtering Task was used to assess attentional control and information processing speed. Each trial showed a horizontal row of five fish. The participant was asked to respond as quickly as possible to	-Flanker processing speed

	whether the central fish was facing to the left or right. Congruent trials were the ones with all five fish in the horizontal row pointing in the same direction and incongruent trials were the ones with the four distracting fishes pointing in the opposite direction of the central target fish. Mean reaction time of the congruent condition was used to assess information processing speed. Higher mean reaction times reflect slower processing speed. The inhibition score (reaction time in incongruent conditions – reaction time in congruent conditions) was used as a measure of attentional control. Higher inhibition scores reflect increased difficulties in attentional control.	-Flanker inhibition
	Reality Filtering Task [5,6]	
	The Reality Filtering task child-adapted version was used to assess recognition memory and orbitofrontal reality filtering. It consisted of a continuous recognition task composed of two runs with the same picture set but arranged in different order. The Temporal Context Confusion index was used as a reality filtering score (TCC as defined by Schnider, 2018): TCC = (FP2/Hits2) – (FP1/Hits1), where FP2 and FP1 are the false positives in run 2 and run 1 (i.e., the incorrect “yes” responses to images that have not yet been presented in the ongoing run), and Hits2 and Hits1 are the correct recognitions of targets in run 2 and run 1 respectively (i.e., the correctly-recognized repetitions). Higher TCC score indicate increased reality filtering difficulties.	Reality filtering TCC
	**Behaviour and socio-emotional competences**	
	Parent questionnaire	
	Strength and Difficulties Questionnaire, parent version (SDQ, Goodman [7])	
	The SDQ parent questionnaire assess overall behaviour problems, emotional symptoms, hyperactivity and inattention, peer relationship problems, and prosocial behaviour. It rates participant's behaviour over the previous 6 months. The SDQ is scored on a Likert scale and includes 25 items, providing a Total Difficulties score. Higher Total Difficulty scores reflect increased behavioural and socio-emotional difficulties.	SDQ total
	Self-reported questionnaires	
	KIDSCREEN-27 [8]	
	The KIDSCREEN-27 is a self-reported questionnaire providing an index of health-related quality of life in children and adolescents. This instrument scored on a Likert scale and includes 27 items, providing a total score. Higher total scores reflect increased quality of life and well-being.	KIDSCREEN total
	Social Goal Scale (SGS, Patrick, et al. [9])	
	The SGS is a self-reported questionnaire providing an index of social responsiveness and of goals setting which ultimately gets you involve with some social work. This instrument scored on a Likert scale and includes 11 items providing a total score. Higher total scores reflect increased social goal in daily life.	Social goal
	Self-Compassion Scale – Short form (SCS, Raes, et al. [10])	
	The SCS is a self-reported questionnaire comprising 12 items, which produces a total score. Higher total scores reflect increased self-compassion.	Self-compassion
	Neuropsychological tests	
	Affect Recognition (NEPSY-II, Korkman, et al. [11]	
	The affect recognition subtest assesses the ability to recognise facial emotional expressions (happy, sad, anger, fear, disgust, and neutral) from photographs of children’s faces in several matching tasks. In the first task, the participant selected one of the four faces that depicted the same emotion as a child's face at the top of the page.	Affect recognition

		In a second task, the participant selected two photographs of faces that displayed the same affect from a selection of four photographs. Finally, the participant examined a photograph of a child's face for 5 seconds, and then from memory, selected two photographs that matched the same emotion as the face previously shown. Standardised scores were used (M=10; SD=3). Higher scores reflect better affect recognition skills.	
		Theory of Mind (NEPSY-II, Korkman, et al. [11])	
		The theory of mind subtest measures understanding of mental functions and other people's perspectives.<br>In the first task, questions are asked to the participant about different verbal scenarios measuring understanding of beliefs, intentions, others' thoughts, ideas and comprehension of figurative language. In the second task, participants have to match facial emotional expressions, from photographs of children's faces, to a scenario. The total raw score was age-adjusted for each participant using the procedure described in the main statistical analyses section. Higher scores reflect better theory of mind skills.	Theory of mind

Supplementary Table S2. Theme addressed during each session of the MBI intervention

Session	SESSION THEME<br>Session Intention<br>Session Mindfulness Attitude	Agenda	Home practice
**1**	**ATTENTION and AUTOPILOT**		
	Introduction to attention and autopilot mode<br>Beginners Mind	Group and instructor introduction<br>Mindfulness definition: *To pay attention to what happens in the present moment, with curiosity and non-judgmentally.*<br>Dialogue about attention awareness and focus of attention<br>The 6 channels: 5 senses and thoughts<br>Practice: Eating a raisin as an explorer<br>Practice: Grounding mediation<br>Closure practice	Chart: Attention - where is my attention now?<br>Practice: Mindful eating – a mindful bite once a day<br>Practice: Grounding meditation
**2**	**DISCOVERING THE BODY LANGUAGE**		
	Discovering bodily sensations<br>Acceptance	Opening practice: Grounding meditation<br>Dialogue about home practice<br>Dialogue about sensations and sensation awareness<br>Practice: Lying down Body scan, with a component of contraction and relaxation at the beginning<br>Practice: Seated body scan<br>Closure practice	Chart: Cool moment of the day<br>Practice: Doing mindfully something habitually done on autopilot<br>Practice: Grounding meditation<br>Practice: Body scan
**3**	**ATTENTION STABILIZATION**		
	Discovering the breath<br>Non-striving	Opening practice: Grounding meditation<br>Dialogue about home practice<br>Dialogue about the breath and it's use as a possible anchor<br>Practice: The 3 minutes' break<br>Practice: Stop and breath<br>Practice: Yoga posture<br>Closure practice	Chart: Bad moment of the day<br>Practice: Body scan<br>Practice: 3 minutes' break<br>Practice: Stop and breath<br>Practice: Yoga posture
**4**	**RECOGNIZING EMOTIONS**		
	Recognizing emotions from bodily sensations	Opening practice: Grounding meditation<br>Dialogue about home practice<br>Practice: Seated body scan, including emotion<br>Dialogue about emotions: recognizing emotions from bodily sensation<br>Practice: Sitting meditation: after remembering a difficult moment, recognize the emotion(s) and it'-s manifestations<br>Drawing and naming the identified emotion<br>Closure practice: The 3 minutes' break	Chart: Emotions - recognizing links between sensations, thoughts and behavior<br>Practice: Yoga posture<br>Practice: The 3 minutes' break<br>Practice: Sitting meditation (emotions)
**5**	**RECOGNIZING THOUGTHS**		
	I'm much more than my thoughts<br>Non-judging	Opening practice: Grounding meditation<br>Dialogue about home practice<br>Walking down the street exercise<br>Discussion about thoughts and emotions	Chart: Cool moment of the day - noting sensations, thoughts and behavior<br>Practice: Sitting meditation (emotions)<br>Practice: Walking mediation

		Practice: walking meditation<br>Closure practice	
**6**	**AUTOMATIC REACTION OR CONSCIOUS RESPONSE?**		
	Exploring stressors and stress reaction<br>Letting go	Opening practice: Grounding meditation<br>Dialogue about home practice<br>Quick board game: identifying stress reaction<br>Discussion about stressors and stress reaction strategies<br>Practice: 5 minutes to deal with stress<br>Closure practice	Chart: Bad moment of the day - noting sensations, thoughts and behavior<br>Chart: Identifying qualities on others<br>Practice: 5 minutes to deal with stress<br>Practice: Walking mediation
**7**	**KINDNESS**		
	Being kind to oneself and to others<br>Gratitude and generosity	Opening practice: Grounding meditation<br>Dialogue about home practice<br>Discussion about kindness and compassion<br>Practice: finding refuge<br>Drawing our refuge<br>Mindful listening exercise<br>Dialogue about communications and social media<br>Closure practice	Letter to oneself:<br>What did I learned and I don't want to forget?<br>What did I learn about myself?<br>Which meditations do I want to keep practicing?<br>Practice: Refuge<br>Practice: Chose another meditation to practice
**8**	**CLOSURE AND OPENNESS**		
	Integrating the program<br>Trust	Opening practice: Grounding meditation<br>Dialogue about home practice<br>Practice: sitting meditation about the program<br>Satisfaction questionnaire<br>How to facilitate our own practice<br>Closing ritual	

Supplementary Table S3. Group comparison on neurobehavioural outcome measures of the VPT and full-term young adolescents.

	Preterms Mean (SD)	Full-terms Mean (SD)	Group comparison	Cohen's d
**BRIEF GEC**	**64.28 (9.66)**	**47.6 (11.09)**	**t(36.31)= 5.541, p<0.001, q<0.001**	**1.63**
**BRIEF MI**	**64.75 (10.39)**	**49.1 (10.64)**	**t(39.74)= 5.209, p<0.001, q<0.001**	**1.49**
**BRIEF BRI**	**61.91 (14.89)**	**47.45 (13.80)**	**t(42.83)= 3.564, p=0.001, q=0.004**	**1.00**
Letter-Number sequencing	10.63 (2.19)	11.24 (2.84)	t(43.04)=-0.996, p=0.325, q=0.505	0.28
Tempo test	16.19 (5.63)	17.95 (2.84)	t(48.4)=-1.506, p=0.139, q=0.277	0.37
Flanker processing speed	998.91 (205.64)	924.54 (183.21)	t(41.51)=1.338, p=0.188, q=0.329	0.38
Flanker inhibition	55.21 (78.93)	19.94 (67.05)	t(42.93)=1.698, p=0.097, q=0.226	0.47
Reality filtering TCC	0.004 (0.01)	0.018 (0.08)	t(19.71)=-0.770, p=0.450, q=0.88	0.28
**SDQ total**	**12.16 (5.14)**	**7.05 (5.91)**	**t(36.25)=3.186, p=0.003, q=0.01**	**0.94**
KIDSCREEN total	106.42 (13.04)	104.74 (15.80)	t(32.74)=0.390, p=0.699, q=0.88	0.12
Social goal	3.8 (0.51)	3.93 (0.43)	t(45.89)=-0.259, p=0.797, q=0.88	0.07
**Self-compassion**	**2.79 (0.47)**	**3.21 (0.54)**	**t(34.36)=-2.821, p=0.008, q=0.022**	**0.85**
Affect recognition	10.38 (2.73)	10.48 (2.09)	t(49.71)=-0.152, p=0.88, q=0.88	0.04
Theory of mind	24.84 (1.71)	24.62 (1.56)	t(45.51)=0.493, p=0.624, q=0.874	0.14

Note: Differences between the VPT and full-term groups were examined using independent-sample t-test. All p-values that survived false discovery rate (FDR) correction (q < 0.05) are indicated in bold.

Supplementary Table S4. Paired-sample t-test on the comparison of neurobehavioural scores before and after MBI intervention in preterm young adolescents.

	Before MBI mean (sd)	After MBI mean (sd)	Paired-sample t-test				
			t	df	d	p	q
**BRIEF GEC**	**64.28 (9.66)**	**58.2 (10.82)**	**4.673**	**29**	**0.854**	**< 0.001**	**< 0.001**
**BRIEF MI**	**64.75 (10.39)**	**56.87 (10.75)**	**3.855**	**29**	**0.682**	**< 0.001**	**< 0.001**
BRIEF BRI	61.91 (14.89)	58.57 (13.22)	1.902	29	0.333	0.067	0.084
**SDQ total**	**12.16 (5.14)**	**10.5 (4.46)**	**2.379**	**29**	**0.434**	**0.024**	**0.040**
Self-compassion	2.79 (0.47)	2.95 (0.6)	-1.632	28	-0.303	0.114	0.114

Note: d shows Cohen's d effect sizes; df degree of liberty; all p-values of paired-sample t-tests that survived false discovery rate (FDR) correction according to Benjamini and Hochberg (1995) are indicated in bold (q < 0.05).

Supplementary Table S5. Mean saliences and their bootstrap-estimated standard deviations for Δ neurobehavioural measures and Δ mean FA of the first PLSC analyses.

**Salience type: Neurobehavioural functioning**	**Salience (bootstrap estimate standard deviation)**
Δ BRIEF GEC	-0.636 (0.073)
Δ BRIEF MI	-0.505 (0.117)
Δ SDQ Total	-0.025 (0.124)
Gestational age	0.062 (0.125)
Age at assessment	-0.457 (0.104)
**Salience type: Δ mean FA on extracted white-matter tracts (TrackSeg)**	**Salience (bootstrap estimate standard deviation)**
Corpus Callosum - Rostrum	0.06 (0.062)
Corpus Callosum - Rostral body	0.103 (0.054)
Corpus Callosum - Anterior midbody	0.092 (0.061)
Corpus Callosum - Posterior midbody	0.087 (0.054)
Corpus Callosum - Splenium	0.034 (0.067)

Corticospinal tract_right	0.092 (0.049)
Inferior cerebellar peduncle_left	0.096 (0.051)
Inferior occipito-frontal fascicle_right	0.044 (0.068)
Inferior longitudinal fascicle_left	0.124 (0.053)
Inferior longitudinal fascicle_right	0.037 (0.067)
Optic radiation_left	0.059 (0.063)
Parieto-occipital pontine_left	0.058 (0.066)
Parieto-occipital pontine_right	0.109 (0.052)
Superior longitudinal fascicle I_left	0.127 (0.045)
Superior longitudinal fascicle I_right	0.089 (0.055)
Superior Thalamic Radiation_left	0.023 (0.056)
Uncinate fascicle_right	0.047 (0.055)
Thalamo-parietal_left	0.083 (0.053)
Thalamo-parietal_right	0.1 (0.049)
Striato-fronto-orbital_right	0.09 (0.048)
Arcuate fascicle_left	0.141 (0.041)
Arcuate fascicle_right	0.129 (0.045)
Anterior Thalamic Radiation_left	0.202 (0.045)
Anterior Thalamic Radiation_right	0.148 (0.043)
Corpus Callosum - Genu	0.164 (0.03)
Corpus Callosum - Isthmus	0.112 (0.049)
Cingulum left	0.138 (0.048)
Cingulum _right	0.137 (0.05)
Corticospinal tract_left	0.14 (0.039)
Fronto-pontine tract_left	0.188 (0.039)
Fronto-pontine tract_right	0.176 (0.046)
Inferior cerebellar peduncle_right	0.123 (0.052)
Inferior occipito-frontal fascicle_left	0.157 (0.052)
Middle cerebellar peduncle	0.156 (0.059)
Optic radiation_right	0.111 (0.051)
Superior cerebellar peduncle_left	0.136 (0.054)
Superior cerebellar peduncle_right	0.177 (0.045)
Superior longitudinal fascicle II_left	0.15 (0.041)
Superior longitudinal fascicle II_right	0.147 (0.035)

Superior longitudinal fascicle III_left	0.159 (0.044)
Superior longitudinal fascicle III_right	0.122 (0.047)
Superior Thalamic Radiation_right	0.109 (0.048)
Uncinate fascicle_left	0.157 (0.05)
Thalamo-premotor_left	0.153 (0.046)
Thalamo-premotor_right	0.158 (0.05)
Thalamo-occipital_left	0.152 (0.051)
Thalamo-occipital_right	0.12 (0.053)
Striato-fronto-orbital_left	0.169 (0.043)
Striato-premotor_left	0.167 (0.036)
Striato-premotor_right	0.118 (0.049)

Supplementary Table S6. Mean saliences and their bootstrap-estimated standard deviations for Δ neurobehavioural measures and Δ mean NDI and ODI of the second PLSC analyses.

**Salience type: Neurobehavioural functioning**	**Salience (bootstrap estimate standard deviation)**
Δ BRIEF GEC	-0.689 (0.068)
Δ BRIEF MI	-0.603 (0.103)
Δ SDQ Total	-0.032 (0.11)
Gestational age	-0.04 (0.08)
Age at assessment	-0.262 (0.099)
**Salience type: Δ mean NDI on extracted white-matter tracts (TrackSeg)**	**Salience (bootstrap estimate standard deviation)**
Arcuate fascicle left	-0.076 (0.032)
Arcuate fascicle right	0.009 (0.038)
Anterior Thalamic Radiation left	-0.098 (0.057)
Anterior Thalamic Radiation right	-0.047 (0.04)
Corpus callusum - Rostrum	-0.029 (0.043)

Corpus callusum -Genu	-0.052 (0.041)
Corpus callusum -Rostral body	-0.014 (0.048)
Corpus callusum -Anterior midbody	0.009 (0.04)
Corpus callusum -Posterior midbody	-0.011 (0.055)
Corpus callusum -Isthmus	-0.031 (0.044)
Corpus callusum -Splenium	-0.035 (0.05)
Cingulum left	-0.046 (0.052)
Cingulum right	-0.023 (0.039)
Corticospinal tract left	-0.039 (0.044)
Corticospinal tract right	0.007 (0.034)
Fronto-pontine tract left	-0.035 (0.037)
Fronto-pontine tract right	-0.003 (0.035)
Inferior cerebellar peduncle left	-0.101 (0.046)
Inferior cerebellar peduncle right	-0.014 (0.045)
Inferior occipito-frontal fascicle left	-0.054 (0.045)
Inferior occipito-frontal fascicle right	0.064 (0.049)
Inferior longitudinal fascicle left	-0.01 (0.049)
Inferior longitudinal fascicle right	0.061 (0.045)
Middle cerebellar peduncle	-0.023 (0.037)
Optic radiation left	-0.086 (0.053)
Optic radiation right	0.005 (0.048)
Parieto-occipital pontine left	-0.006 (0.044)
Parieto-occipital pontine right	0.036 (0.049)
Superior cerebellar peduncle left	-0.018 (0.038)
Superior cerebellar peduncle right	0.031 (0.045)
Superior longitudinal fascicle I left	-0.063 (0.041)
Superior longitudinal fascicle I right	-0.03 (0.04)
Superior longitudinal fascicle II left	-0.066 (0.036)
Superior longitudinal fascicle II right	0.018 (0.035)
Superior longitudinal fascicle III left	-0.026 (0.044)
Superior longitudinal fascicle III right	0.041 (0.045)
Superior Thalamic Radiation left	-0.072 (0.046)
Superior Thalamic Radiation right	-0.014 (0.034)
Uncinate fascicle left	-0.052 (0.049)

Uncinate fascicle right	-0.018 (0.036)
Thalamo-premotor left	-0.039 (0.052)
Thalamo-premotor right	-0.013 (0.057)
Thalamo-parietal left	-0.12 (0.047)
Thalamo-parietal right	0.014 (0.051)
Thalamo-occipital left	-0.103 (0.036)
Thalamo-occipital right	0.007 (0.043)
Striato-fronto-orbital left	-0.028 (0.044)
Striato-fronto-orbital right	0.001 (0.035)
Striato-premotor left	-0.08 (0.045)
Striato-premotor right	-0.032 (0.053)
**Salience type:<br>Δ mean ODI on extracted white-matter tracts (TrackSeg)**	**Salience (bootstrap estimate standard deviation)**
Arcuate fascicle left	-0.142 (0.029)
Arcuate fascicle right	-0.108 (0.037)
Anterior Thalamic Radiation left	-0.157 (0.025)
Anterior Thalamic Radiation right	-0.136 (0.031)
Corpus callusum - Rostrum	-0.099 (0.034)
Corpus callusum -Genu	-0.137 (0.023)
Corpus callusum -Rostral body	-0.08 (0.041)
Corpus callusum -Anterior midbody	-0.051 (0.054)
Corpus callusum -Posterior midbody	-0.067 (0.044)
Corpus callusum -Isthmus	-0.093 (0.054)
Corpus callusum -Splenium	-0.027 (0.059)
Cingulum left	-0.119 (0.037)
Cingulum right	-0.106 (0.032)
Corticospinal tract left	-0.155 (0.03)
Corticospinal tract right	-0.07 (0.04)
Fronto-pontine tract left	-0.186 (0.022)
Fronto-pontine tract right	-0.145 (0.033)
Inferior cerebellar peduncle left	-0.123 (0.049)
Inferior cerebellar peduncle right	-0.135 (0.052)

Inferior occipito-frontal fascicle left	-0.125 (0.05)
Inferior occipito-frontal fascicle right	-0.067 (0.064)
Inferior longitudinal fascicle left	-0.107 (0.046)
Inferior longitudinal fascicle right	-0.012 (0.054)
Middle cerebellar peduncle	-0.166 (0.047)
Optic radiation left	-0.093 (0.052)
Optic radiation right	-0.093 (0.055)
Parieto-occipital pontine left	-0.031 (0.05)
Parieto-occipital pontine right	-0.077 (0.06)
Superior cerebellar peduncle left	-0.117 (0.044)
Superior cerebellar peduncle right	-0.182 (0.038)
Superior longitudinal fascicle I left	-0.104 (0.036)
Superior longitudinal fascicle I right	-0.077 (0.041)
Superior longitudinal fascicle II left	-0.129 (0.031)
Superior longitudinal fascicle II right	-0.121 (0.029)
Superior longitudinal fascicle III left	-0.148 (0.027)
Superior longitudinal fascicle III right	-0.083 (0.044)
Superior Thalamic Radiation left	-0.138 (0.03)
Superior Thalamic Radiation right	-0.11 (0.041)
Uncinate fascicle left	-0.137 (0.028)
Uncinate fascicle right	-0.102 (0.034)
Thalamo-premotor left	-0.135 (0.027)
Thalamo-premotor right	-0.102 (0.032)
Thalamo-parietal left	-0.132 (0.043)
Thalamo-parietal right	-0.105 (0.054)
Thalamo-occipital left	-0.11 (0.039)
Thalamo-occipital right	-0.086 (0.051)
Striato-fronto-orbital left	-0.033 (0.04)
Striato-fronto-orbital right	-0.078 (0.051)
Striato-premotor left	-0.152 (0.039)
Striato-premotor right	-0.072 (0.049)

Supplementary Figures

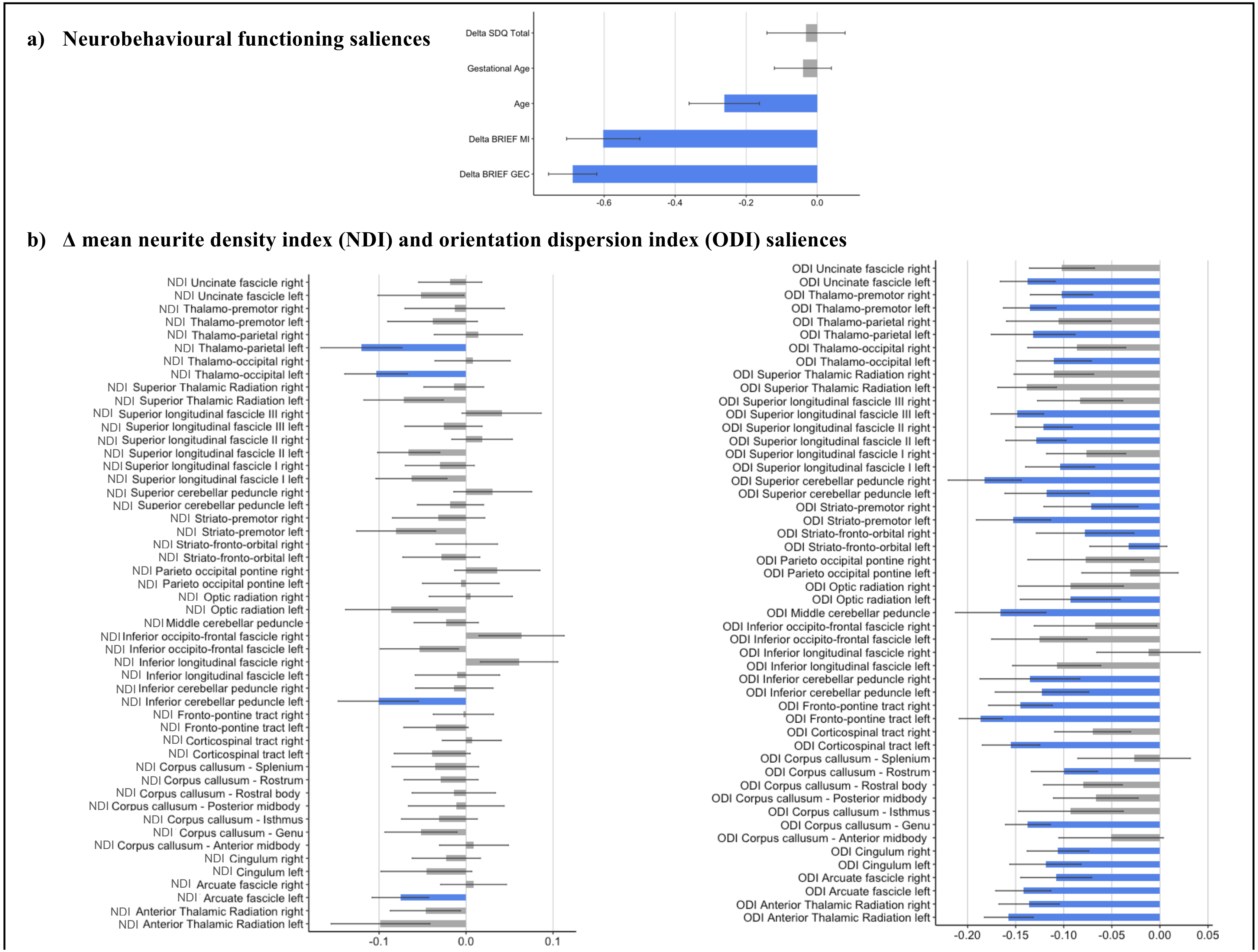

Figure S1. Illustration of the results from the second PLSC analyses. a) Neurobehavioural functioning saliences: the diverging graph show mean saliences averaged across bootstrap samples and their bootstrap-estimated standard deviations (x-axis) for each neurobehavioural functioning measure (y-axis); robust saliences are represented in blue; of note, saliences bellow 0 indicate a decreased in scores after MBI and saliences above 0 indicate an increased in scores after MBI. b) Δ mean neurite density index (NDI) and orientation dispersion index (ODI) saliences: the diverging graph show mean saliences averaged across bootstrap samples and their bootstrap-estimated standard deviations (x-axis) for each Δ mean NDI and ODI along a given tract (y-axis); the tracts extracted from TractSeg and showing robust Δ mean NDI or ODI saliences are represented in blue; of note, saliences below 0 indicate a decreased in scores after MBI and saliences above 0 indicate an increased in scores after MBI.

Supplementary References

1 Gioia, G., Isquith, P., Guy, S. & Kenworthy, L. *BRIEF – Behavior Rating Inventory of Executive Function. Professional manual*. (Psychological Assessment Resources Inc, 2000).

2 Wechsler, D. *Manual for the Wechsler Intelligence Scale for Children-IV*. (Psychological Corporation, 2003).

3 De Vos, T. *TTR: Tempotest rekenen*. (Swets & Zeitlinger, 1992).

4 Christ, S. E., Kester, L. E., Bodner, K. E. & Miles, J. H. Evidence for selective inhibitory impairment in individuals with autism spectrum disorder. *Neuropsychology* **25**, 690-701, doi:10.1037/a0024256 (2011).

5 Liverani, M. C. *et al.* Children's sense of reality: The development of orbitofrontal reality filtering. *Child neuropsychology : a journal on normal and abnormal development in childhood and adolescence* **23**, 408-421, doi:10.1080/09297049.2015.1120861 (2017).

6 Schnider, A. *The confabulating mind : how the brain creates reality*. Second edition. edn, (Oxford University Press, 2018).

7 Goodman, R. Psychometric properties of the strengths and difficulties questionnaire. *J Am Acad Child Adolesc Psychiatry* **40**, 1337-1345, doi:10.1097/00004583-200111000-00015 (2001).

8 Robitail, S. *et al.* Testing the structural and cross-cultural validity of the KIDSCREEN-27 quality of life questionnaire. *Qual Life Res* **16**, 1335-1345, doi:10.1007/s11136-007-9241-1 (2007).

9 Patrick, H., Hicks, L. & Ryan, A. M. Relations of perceived social efficacy and social goal pursuit to self-efficacy for academic work. *Journal of Early Adolescence*, 109-128, doi:10.1177/0272431697017002001 (1997).

10 Raes, F., Pommier, E., Neff, K. D. & Van Gucht, D. Construction and factorial validation of a short form of the Self-Compassion Scale. *Clin Psychol Psychother* **18**, 250-255, doi:10.1002/cpp.702 (2011).

11 Korkman, M., Kirk, U. & Kemp, S. *NEPSY-II: A developmental neuropsychological assessment, second edition*. (2007).
